# Mechanism of interpleat channel blockage in pleated filters during dust loading

**DOI:** 10.1038/s41598-026-51067-z

**Published:** 2026-04-29

**Authors:** Guangping Teng, Caijun Zhao, Guangli Li, Weile Geng

**Affiliations:** 1 School of Safety and Management Engineering, Hunan Institute of Technology, Hengyang, 421002 China; 2 Key Laboratory of Gas and Fire Control for Coal Mines, University of Mining and Technology, Ministry of Education, Xuzhou, 221116 China

**Keywords:** Pressure drop, Pleat ratio, Dust dendrites, Interpleat channel blockage, Engineering, Materials science, Physics

## Abstract

Pleated filters are widely employed in industrial and domestic applications owing to their high dust-holding capacity and compact structure. However, the formation of dust dendrites can lead to blockage of the pleat channels, thereby causing a rapid increase in pressure drop. This study systematically investigates the filtration performance of pleated filters with different pleat ratios and the factors influencing channel blockage through experimental research and theoretical analysis. The results demonstrate that when the dust deposition is substantial, the formation of a large number of dust dendrites can result in interpleat channel blockage and a sudden increase in filter core pressure drop. The critical areal dust loading for channel blockage is identified, and it is revealed that the formation of dust dendrites is influenced by the pleat ratio of the filter, dust particle size, and filtration face velocity. Specifically, higher pleat ratios, smaller dust particle sizes, and filtration face velocities are found to be more conducive to the formation of dust dendrites. Further mechanistic analysis illustrates that dust dendrite formation arises from the dynamic interplay between the combined interparticle forces (van der Waals forces, electrostatic forces, and liquid bridge bonding forces) and the aerodynamic force imposed by the airflow. A strong aerodynamic force causes dendrite fragmentation, while a weak aerodynamic force enables sustained dendrite growth. These results provide valuable technical support for the design and optimization of pleated filter elements in practical engineering.

## Introduction

With the rapid advancement of urbanization and industrialization, particulate matter (PM) pollution has become a growing concern, posing significant threats to both the ecological environment and public health. This issue has garnered widespread attention from society. The sources of PM pollution are diverse, including industrial emissions, fossil fuel combustion, natural dust, and vehicle exhaust, making it challenging to control and prevent^1–3^. To combat this pollution, dry filtration technology for dust removal has been continuously refined. Through the upgrading of filter materials and the optimization of equipment structures, dust removal performance has been significantly improved. As a result, this technology has been widely applied in both industrial and domestic settings. Among various dry dust removal systems, pleated filters have become increasingly popular due to their large filtration area, high dust holding capacity, and compact structure^4,5^.

To enhance the filtration performance of pleated filters, numerous scholars have conducted research on filtration velocity, dust deposition patterns, effective filtration area, and optimal pleat ratio. Subrenat et al.^6^ discovered through numerical simulation that the filtration velocity is close to zero in the pleated areas due to the overlap of filter media, rendering these areas ineffective for filtration and thus classifying them as dead zones. Li et al.^4^ conducted numerical simulations to study the impact of pleat structure on airflow distribution, pressure drop, and particle deposition characteristics. They discovered that pleating significantly affects filtration velocity, with higher pleat ratios leading to a greater tendency for dust particles to deposit at the pleat bottoms. This reduces the effective filtration area and increases the pressure drop. Teng et al.^7^ found that pleating decreases the effective filtration area, and the uneven deposition of dust further reduces the effective filtration area as the amount of dust deposition increases. Yit et al.^5^ and Persaud et al.^2^ respectively conducted experimental and numerical studies on the influence of pleat geometry on filtration performance and determined the optimal pleat ratio range.

During the dust deposition process, particles form dendritic structures that can block interpleat channels, causing a rapid pressure drop increase. Hong et al.^1^ used scanning electron microscopy to capture images of ultrafine fibers capturing dust particles and forming dendrites. Kasper et al.^8^ employed confocal laser scanning microscopy to image dust deposition-formed dendrites. They noted that most particles accumulate on existing dendrites, rapidly lengthening and broadening them. Fotovati et al.^9^ experimentally observed that at low air velocities, dust particles form chain-like accumulations called dust dendrites in the upper part of pleat channels, while significant dendrite formation is difficult at higher velocities. Bourrous et al.^10,11^ used high-speed cameras to study dust particle deposition on pleated filters, observing dendritic structure formation within pleated channels and noting a relationship with wind speed. Saleh et al.^12^ reported from experimental investigations that blockage of the interpleat channels by dust dendrites arises under conditions of small interpleat spacing and low filtration face velocity. Therefore, further research into the governing factors and formation mechanisms of dust dendrites is therefore warranted to preclude interpleat channel blockage caused by dust dendrite development during the structural design and specification of pleated filter elements.

In this paper, dust filtration experiments were carried out to investigate the dynamic variation laws of pressure drop for pleated filters with different pleat ratios. The critical areal dust loading for the onset of interpleat channel blockage was determined, the influencing factors governing interpleat channel blockage were analyzed, and the mechanisms associated with dust dendrite formation were elucidated.

## Experiments

### Experimental system

**Fig. 1:**
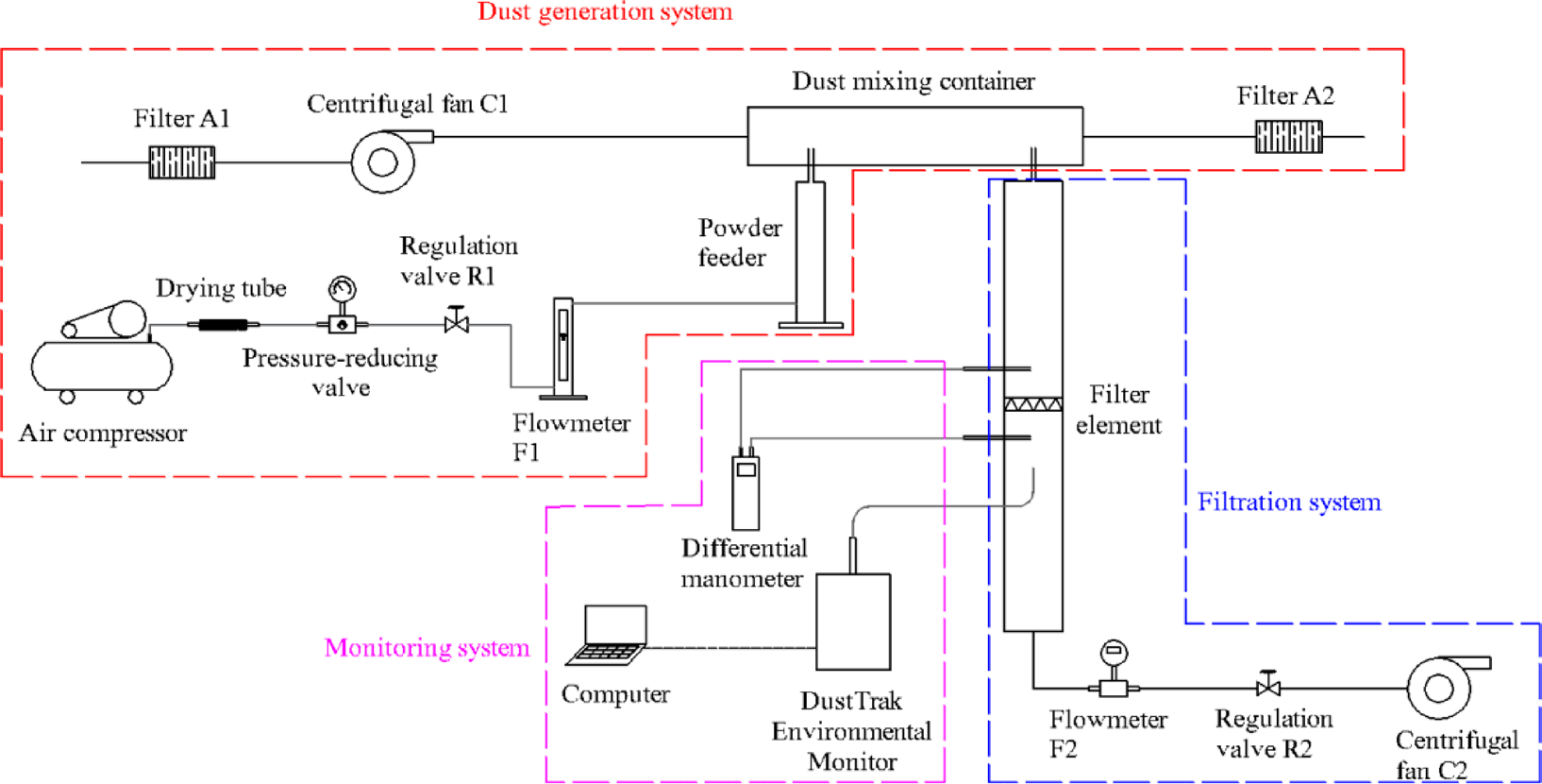
Schematic diagram of the experimental system.

The dust filtration experimental system, as illustrated in Fig. 1, has been comprehensively described in a previous study^7^. It is composed of three integral parts: the dust generation system, the filtration system, and the monitoring system. The high-pressure gas from the air compressor is first dried and regulated by a pressure reducing valve to form a constant low-pressure gas. The gas flow rate is then adjusted via regulation valve R1 to control the feeding rate of the powder feeder, thereby regulating the dust concentration. The filtration system features adjustable air volume, facilitating dust filtration experiments across a spectrum of airflow conditions. The monitoring system is capable of real-time measurements of dust concentration upstream and downstream of the filter element, as well as the pressure drop across the filter element.

### Experiment materials

**Fig. 2:**
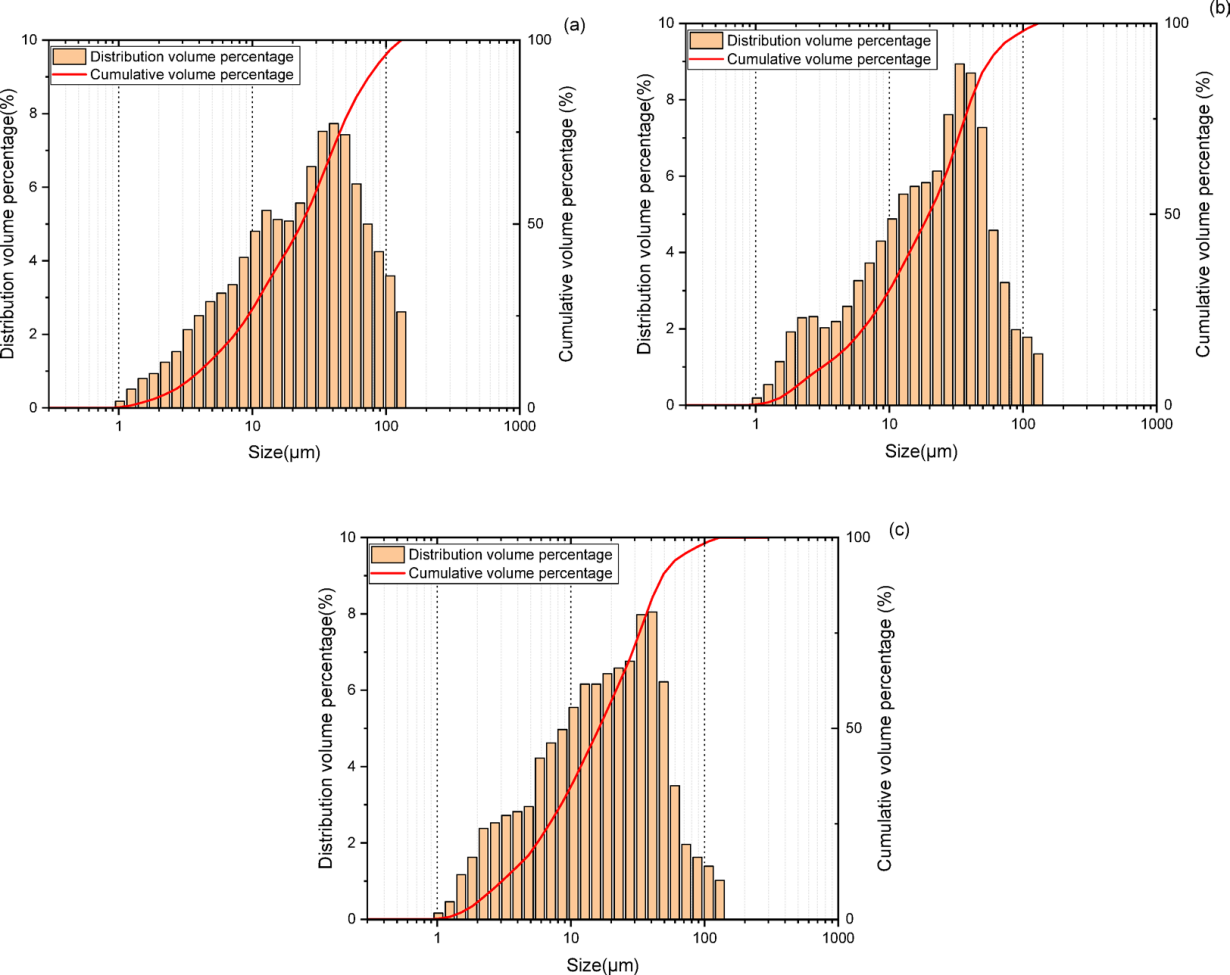
Dust particle size distribution **a** 200-mesh fly ash, **b** 400-mesh fly ash, **c** 600-mesh fly ash.

The dust utilized in this experiment was fly ash with distinct particle size distributions, which was obtained through repeated sieving with 200-mesh, 400-mesh, and 600-mesh screens. Subsequently, the dust was subjected to drying at a constant temperature of 100 °C for 5 h in a drying oven to eliminate moisture. The particle size distribution of the dust was characterized using a laser particle size analyzer (winner2000, Jinan Winner Particle Instrument Stock Co., Ltd., Jinan, China), as depicted in Fig. 2. The mean and median particle sizes of the dust are summarized in Table 1.

**Table 1:** Particle size test data of fly ash.

Parameter	200-mesh	400-mesh	600-mesh
Mean particle size(µm)	31.45	25.89	22.49
Median particle size(µm)	23.34	19.76	15.23

**Fig. 3:**
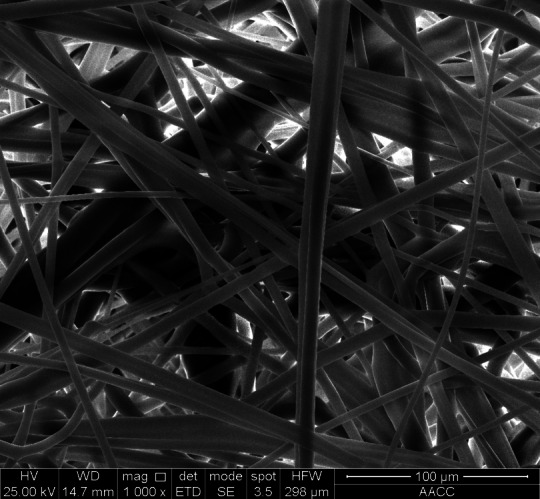
SEM image of the filter media surface at 1000× magnification.

The filter media employed in this study was polypropylene ultrafine fiber material (Henan Aklly Filter Engineering Co., Ltd., Xinxiang, China), with an E10 filtration efficiency rating, a thickness of 0.5 mm, and a basis weight of 110 g/m². Figure 3 displays the scanning electron microscopy (SEM) image of the filter surface at 1000× magnification, revealing a multi-layered random fibrous arrangement. Statistical analysis of the fiber diameters in the SEM images indicates that the mean fiber diameter of the filter media is approximately 6.3 μm.

**Fig. 4:**
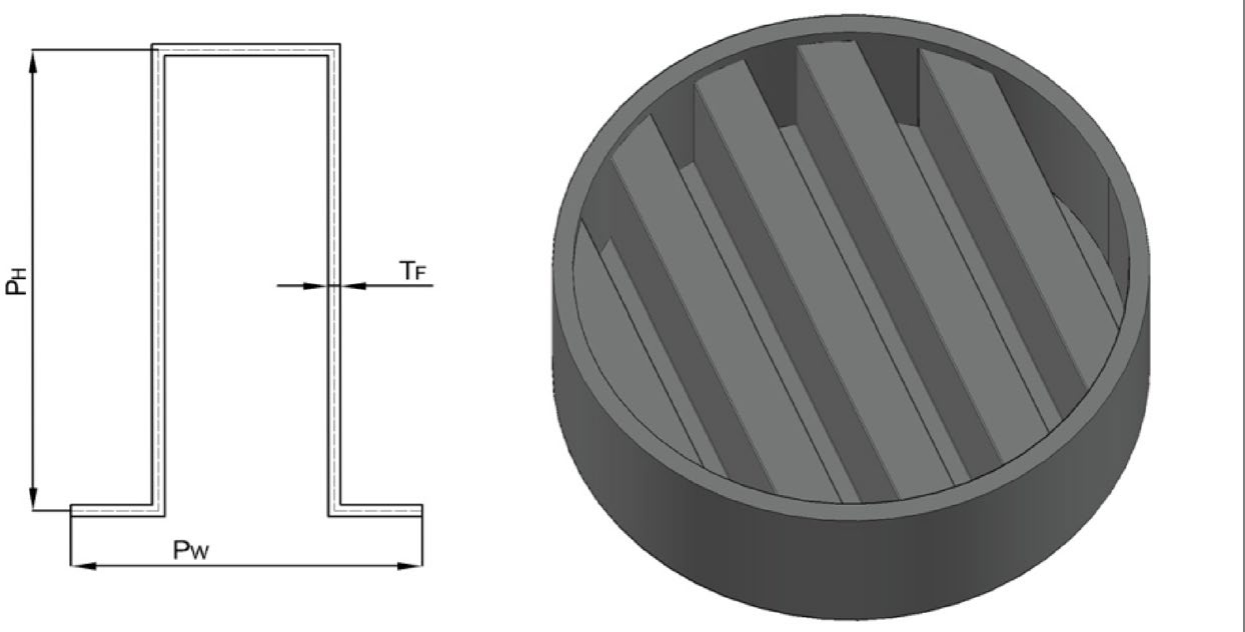
Geometric structure of the pleats.

In the experiment, self-made pleated filter cartridges were utilized. Each cartridge utilized a transparent polymethyl methacrylate ring as the outer frame, with an outer diameter of 150 mm, an inner diameter of 140 mm, and a height of 25 mm. The geometric structure of the pleats is illustrated in Fig. 4. The self-made filter cartridges were fabricated with pleat numbers of 5, 10, 15, 20, 25, 30, 35, and 40 respectively, and a pleat height of 20 mm. The pleated media was fixed and sealed to the outer frame using hot-melt adhesive, yielding an effective filtration diameter of approximately 135 mm. Detailed parameters of the self-made filter cartridges are listed in Table 2, where the filter cartridge with zero pleats is defined as having a pleat ratio of 0.

**Table 2:** Parameters of the pleated filter cartridges.

Parameter	α0	α0.71	α1.43	α2.14	α2.86	α3.57	α4.29	α5.00	α5.71
Number of pleats/N	/	5	10	15	20	25	30	35	40
Pleat height/P_h_(mm)	/	20	20	20	20	20	20	20	20
Pleat pitch/P_w_(mm)	/	13.50	6.50	4.17	3.00	2.30	1.83	1.50	1.25
Pleat ratio/α = P_h_/P_w_	0	0.71	1.43	2.14	2.86	3.57	4.29	5.00	5.71
Filtration area/S(cm^2^)	143	327	531	736	940	1144	1349	1553	1758

### Experimental procedure

In a dust-free air flow, the filtration flow rate was adjusted using flow control valve R2 to vary the filtration face velocity within the range of 1–7 cm/s, and the pressure drop of filter cartridges with different pleat ratios was recorded. During the experiment, the temperature was controlled at 25 ± 3 °C using an air conditioner, while the relative humidity (RH) was maintained at 75 ± 5% with a dehumidifier and a humidifier. For each test, the pressure drop was calculated as the average of instantaneous values recorded at 10‑second intervals over a 1‑minute period. All measurements were repeated in triplicate using independent filter cartridges to ensure the relative error was within 5%. The pressure drop of the cartridges was determined as the average of the three replicate runs.

For the dust filtration experiments, the dust feed rate of the dust generator was adjusted to stabilize the dust concentration in the dust mixing chamber at approximately 800 ± 40 mg/m³, with a relative error not exceeding 5%. The test dust employed was fly ash of 200, 400 and 600 mesh, respectively. Filtration flow rate was varied by regulating valve R2 to maintain the filtration face velocity at steady values of 1, 2 and 4 cm/s for separate test runs. During the experiment, the temperature was controlled at 25 ± 3 °C using an air conditioner, while the RH was maintained at 75 ± 5% with a dehumidifier and a humidifier. The filtration duration was controlled to achieve the areal dust loading of 5, 10, 15, 20 and 25 mg/cm², and the experimental durations are presented in Table 3. The pressure drop under different experimental conditions was measured in real time using an AP800 differential pressure gauge (TSI, USA), and the instantaneous pressure drop data at the moment of the completion of each experiment were recorded. The filter cartridges were weighed both before and after each test to calculate the actual areal dust loading. Any experiment with a measurement error exceeding 5% was deemed invalid and had to be repeated. A brand-new, unused filter cartridge was employed for each individual experiment, and all tests were performed in triplicate to guarantee the repeatability and reliability of the experimental results.

**Table 3:** Dust filtration times under different experimental conditions.

Face velocity (cm/s)	Areal dust loading(mg/cm²)				
	5	10	15	20	25
	Time				
1	1 h 44 min	3 h 28 min	5 h 12 min	6 h 56 min	8 h 40 min
2	52 min	1 h 44 min	2 h 36 min	3 h 28 min	4 h 20 min
4	26 min	52 min	1 h 18 min	1 h 44 min	2 h 10 min

## Results and analysis

### Effect of pleat ratio on initial pressure drop of filter cartridges

**Fig. 5:**
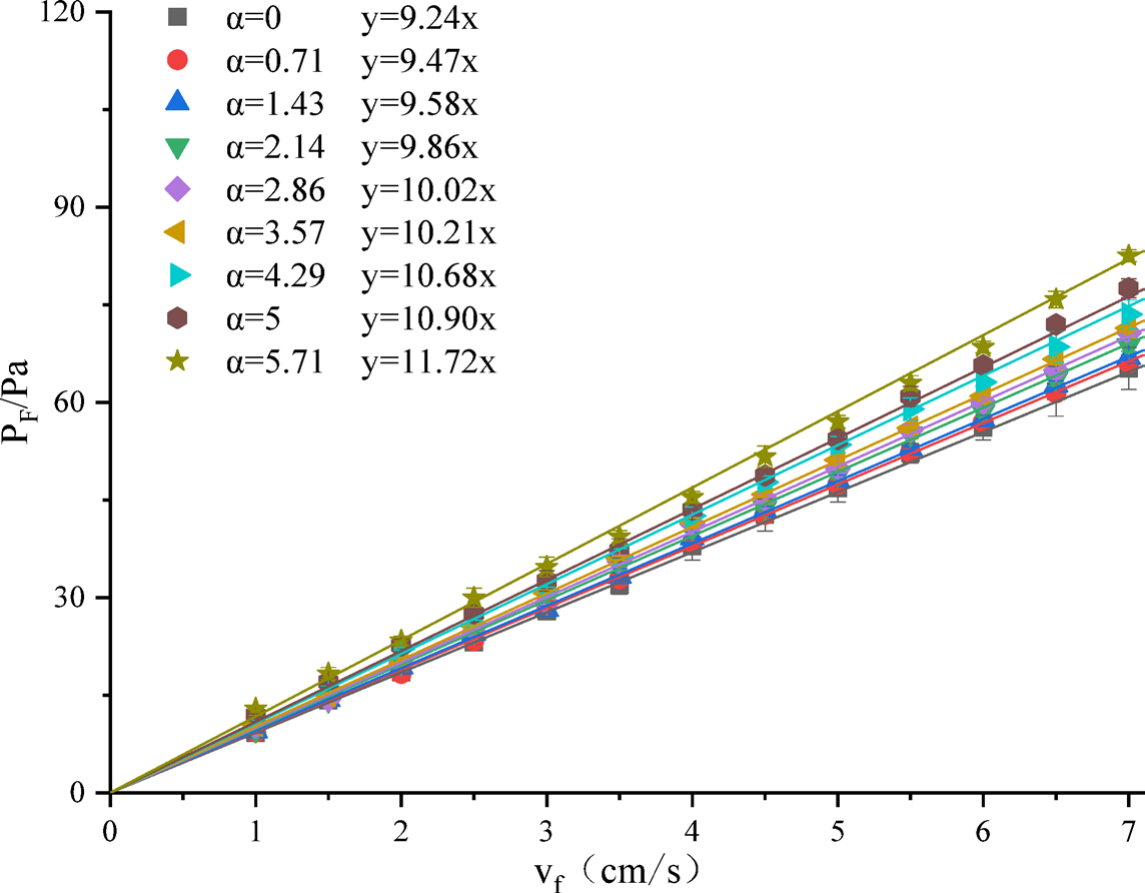
Variation of initial pressure drop of filter cartridges (${P_F}$) with filtration face velocity (${\nu _f}$).

Figure 5 shows the initial pressure drop of the filter element. It can be observed from the figure that the pressure drop of the filter cartridge increases linearly with filtration face velocity, and the greater the pleat ratio, the faster the pressure drop increases. Linear fitting was performed on the experimental data to obtain the fitting equation, where the slope of the fitted line represents the drag coefficient of the filter cartridge, which ranges from 9.24 to 11.72 Pa·s·cm⁻¹. The fitting equation further indicates that the drag coefficient increases with an increase in pleat ratio.

Studies have demonstrated that the increase in the filter cartridge drag coefficient with rising pleat ratio mainly arises from two factors: the reduction in effective filtration area and the increase in structural resistance^4,7,13–15^. Phenomena such as deformation, compression and overlapping occur at the bends of the filter media, which reduce the permeability of the media^6^. Furthermore, previous numerical simulations have revealed that a larger pleat ratio corresponds to a larger ineffective filtration zone near the pleat corners, which in turn gives rise to a decrease in the effective filtration area^14^. Measurements of the flow velocity within the pleated structure indicate that pleating induces non-uniform velocity distribution in the interpleat channels. A larger pleat ratio exacerbates the velocity discrepancy, accompanied by higher wall shear stress and steeper velocity gradients, thereby elevating the pressure loss^4^.

### Effect of pleat ratio on pressure drop of filters during dust loading

**Fig. 6:**
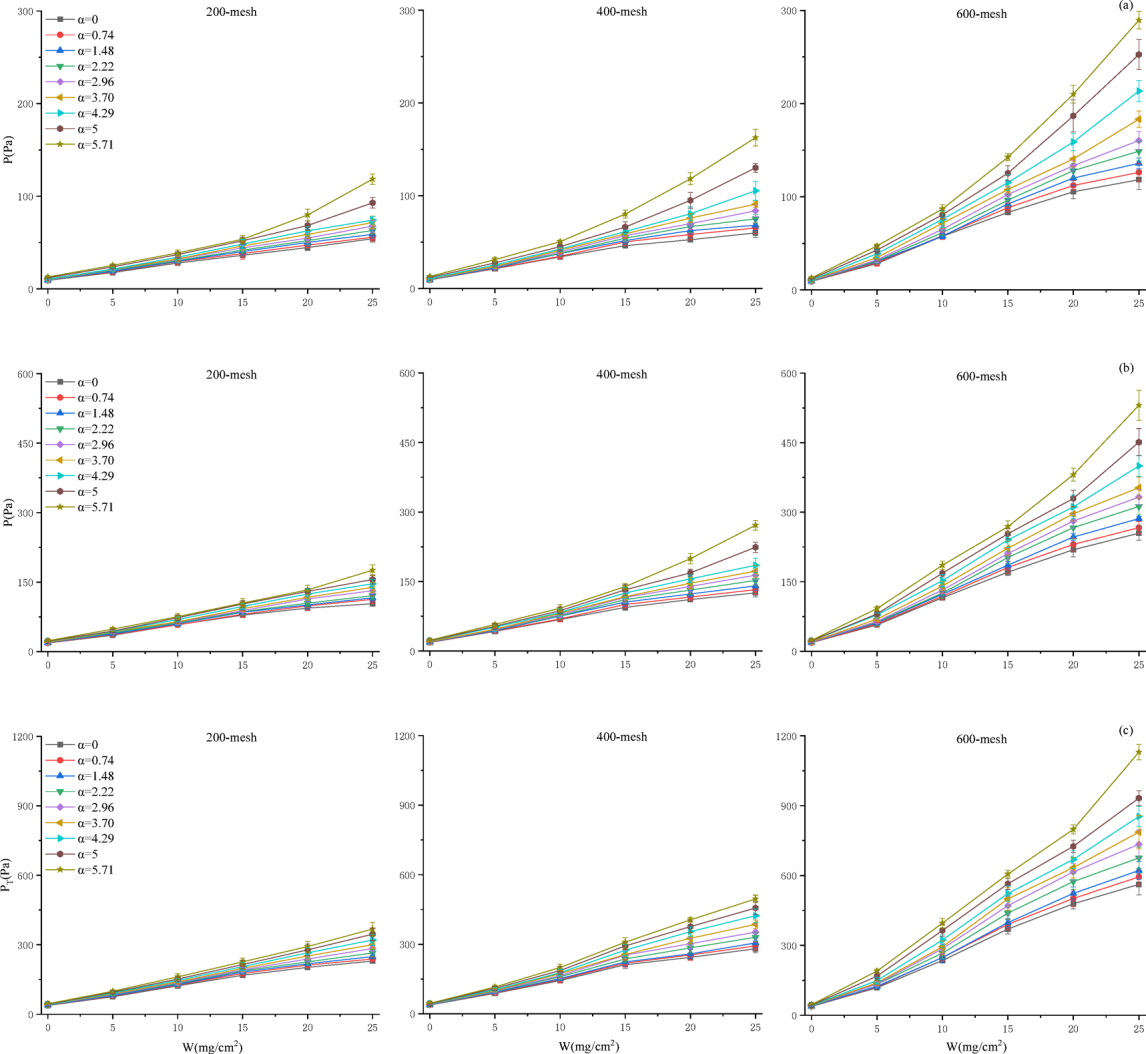
Variation of pressure drop of filter cartridge (${P_T}$) with areal dust loading (*W*). **a** ${v_f}$=1 cm/s, **b** ${v_f}$=2 cm/s, **c** ${v_f}$=4 cm/s.

Figure 6 presents the variation in filter cartridge pressure drop with areal dust loading at a dust concentration of 800 ± 40 mg/m³ and filtration face velocities of 1, 2, and 4 cm/s, respectively. It can be observed that the pressure drop shows a consistent increasing trend with rising areal dust loading under all test conditions. The total pressure drop across the filter comprises two components: the pressure drop of the filter media and that induced by the dust cake. As the dust cake pressure drop is proportional to its thickness, it increases correspondingly with areal dust loading.

A comparison of pressure drop evolution across different particle sizes reveals that the pressure drop rises more rapidly with finer dust particles. Our previous results demonstrated that finer particles generate a denser dust cake with lower porosity, thereby promoting a more rapid increase in the overall filter pressure drop^7^. These findings are in good agreement with those reported by Li et al.^16^, Wang et al.^17^, Hennemann et al.^18^, and Alotoom et al.^19^.

When comparing the pressure drop of filter cartridges with different pleat ratios, it was found that the pressure drop increases with the pleat ratio at the same areal dust loading. This trend aligns with the variation in the initial pressure drop of filter cartridges presented in the section "Effect of pleat ratio on initial pressure drop of filter cartridges", and is ascribed to the diminished effective filtration area and elevated structural flow resistance. Previous work has confirmed heterogeneous dust deposition on pleated media: heavy accumulation at pleat roots further shrinks the effective filtration area, with greater area loss at higher pleat ratios^7,14^. Meanwhile, the development of a dust cake further constricts the interpleat flow channels, thereby increasing interpleat air velocity and augmenting the structural flow resistance^15^.

### Study on influencing factors of dust clogging

It can also be observed from Fig. 6 that at low pleat ratios and low areal dust loading, the pressure drop rises slowly and nearly linearly with increasing areal dust loading. By contrast, when these two parameters are sufficiently high, the pressure drop first exhibits linear growth and then rises at an accelerated rate with increasing areal dust loading. This behavior arises from the onset of interpleat channel clogging within the filter cartridge during dust filtration tests.

**Fig. 7:**
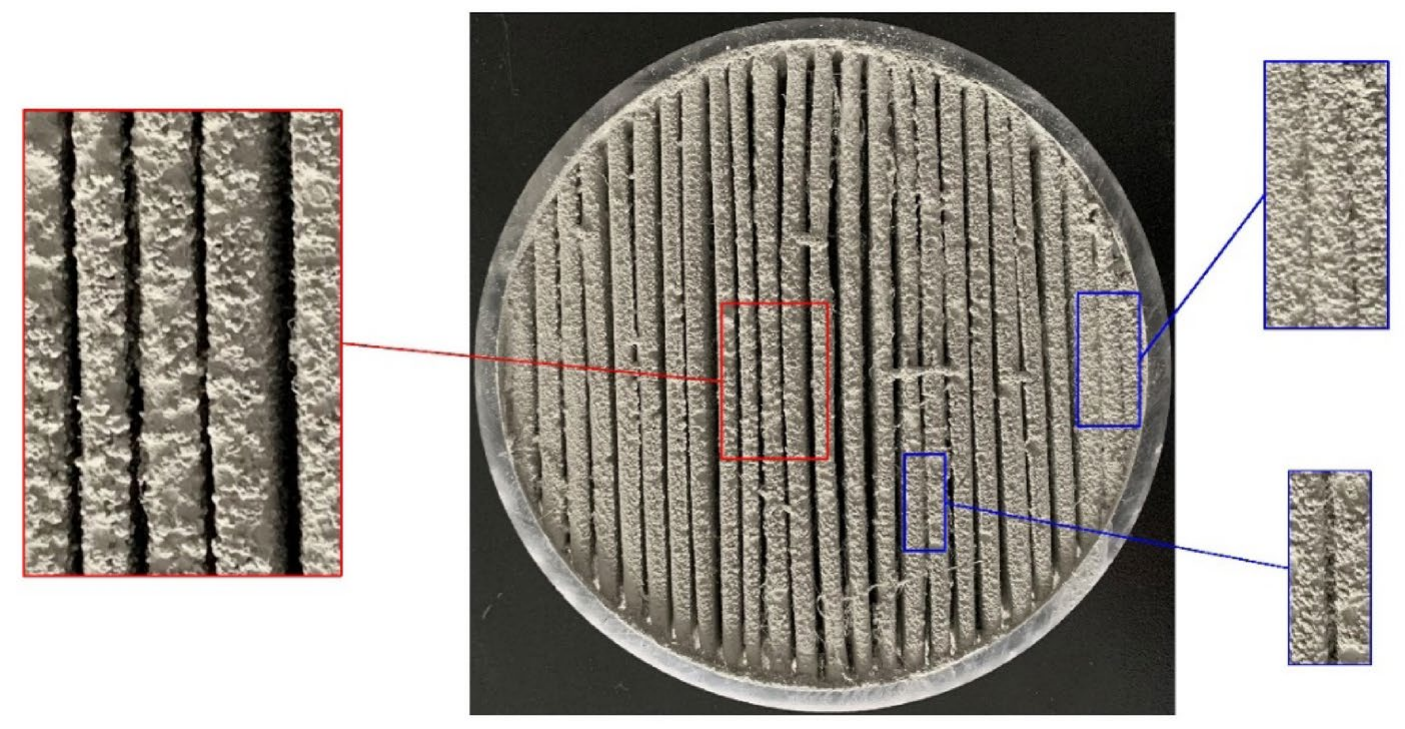
Physical image of dust deposition on pleated filter cartridges.

Figure 7 shows the physical image of dust deposition with a pleat ratio of 3.7, filtration face velocity of 1 cm/s, 600-mesh fly ash, and areal dust loading of 10 mg/cm². As shown in the red-boxed region, dust deposition on the filter surface is uneven and heterogeneous, with particles accumulating in clusters to form dust dendrites through chain-like aggregation. As indicated in the blue-boxed area, local blockage takes place at the crests of certain inter-pleat channels within the pleated filter cartridge.

Driven by interparticle forces (van der Waals attraction, electrostatic adhesion, and liquid bridge bonding), dust particles agglomerate into chain-like dendritic structures on the filter surface, as illustrated by the protruding particulate deposits on the filter media in Fig. 7. At heavy areal dust loading, dust deposition results in clogging of the filter interpleat channels, as illustrated by the blue regions in Fig. 7. These dendritic morphologies trigger premature interpleat channel blockage, leading to a marked rise in pressure drop with continued dust deposition^8,10,12,20^.

**Fig. 8:**
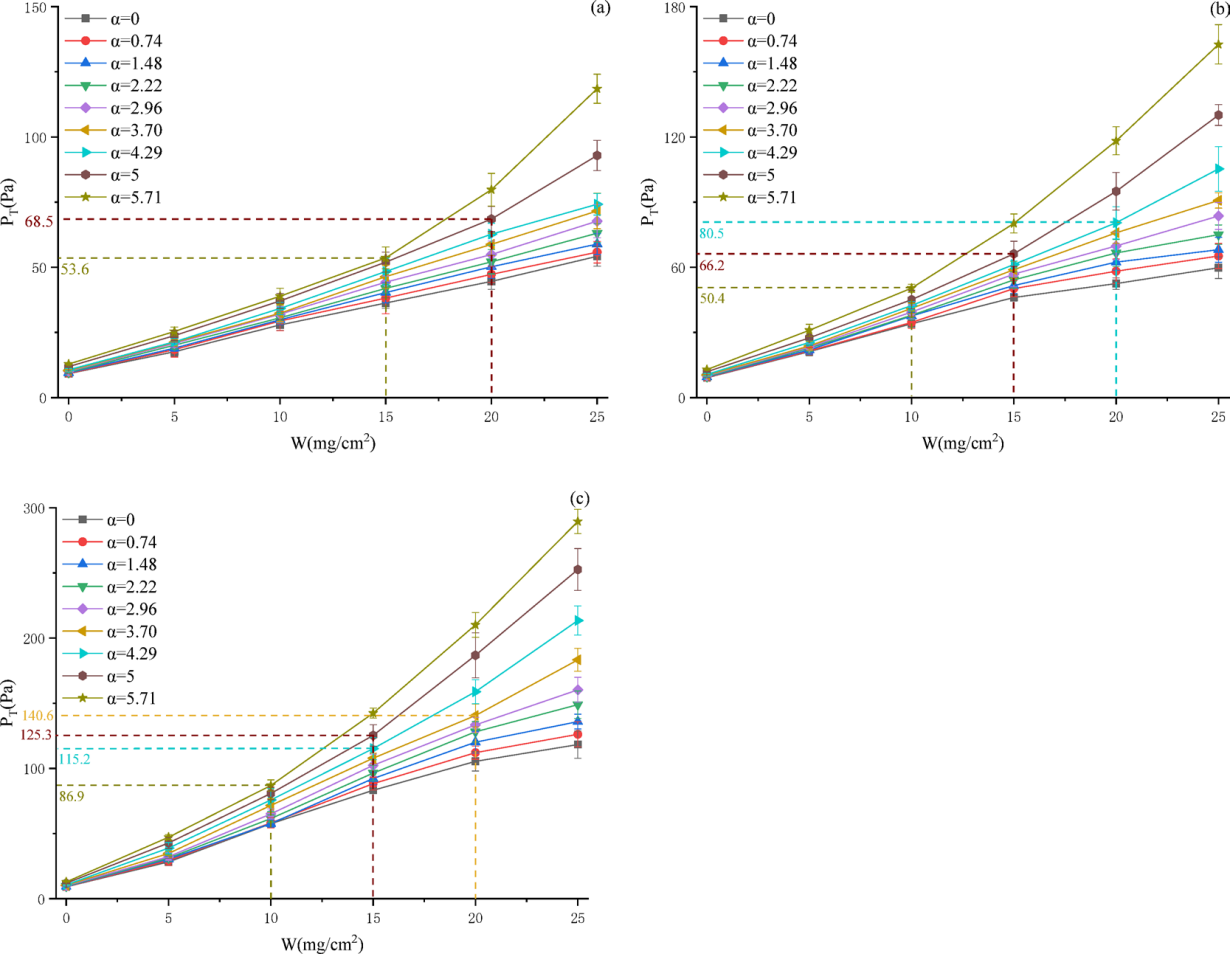
Turning points of the filter pressure drop at a filtration face velocity of 1 cm/s **a** 200-mesh fly ash, **b** 400-mesh fly ash, **c** 600-mesh fly ash. The dashed lines and corresponding values represent the locations of the pressure drop turning points and the associated pressure drops.

**Fig. 9:**
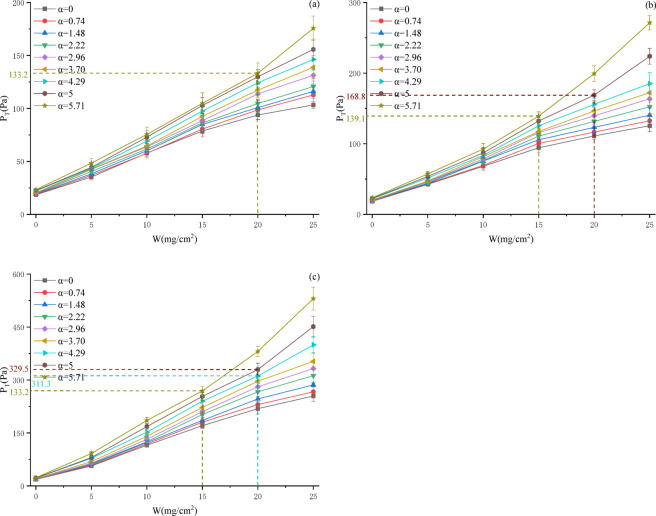
Turning points of the filter pressure drop at a filtration face velocity of 2 cm/s **a** 200-mesh fly ash, **b** 400-mesh fly ash, **c** 600-mesh fly ash.

**Fig. 10:**
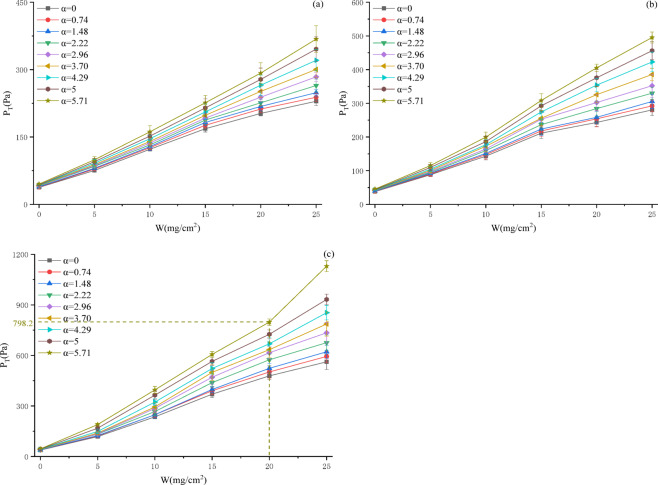
Turning points of the filter pressure drop at a filtration face velocity of 4 cm/s **a** 200-mesh fly ash, **b** 400-mesh fly ash, **c** 600-mesh fly ash.

Dendritic dust structures gradually form as dust particles deposit, and their continued growth progressively clogs the interpleat channels. Once a considerable area of these channels becomes blocked, the pressure drop across the filter element rises sharply, yielding a distinct turning point for this rapid increase. Figures 8, 9 and 10 present the turning points of the filter pressure drop at filtration face velocities of 1, 2, and 4 cm/s, respectively.

Figure 8 shows the turning points of filtration pressure drop for various particle sizes at a filtration face velocity of 1 cm/s. During filtration of 200-mesh fly ash, an abrupt rise in pressure drop occurs at pleat ratios of 5 and 5.71, with the corresponding areal dust loadings at the turning point being 20 and 15 mg/cm², respectively. For 400-mesh fly ash, the areal dust loadings at the pressure drop turning points are 20, 15, and 10 mg/cm² at pleat ratios of 4.29, 5, and 5.71, respectively. For 600-mesh fly ash, the corresponding values are 20, 15, 15, and 10 mg/cm² at pleat ratios of 3.70, 4.29, 5, and 5.71, respectively. The turning points of the filter pressure drop at filtration velocities of 2 and 4 cm/s can be obtained using the same approach, as shown in Figs. 9 and 10, respectively.

In the experiment, filter pressure drop was measured at every 5 mg/cm² increment in areal dust loading. As a result, the pressure drop turning points in Figs. 8, 9 and 10 are approximate locations where the pressure drop increases abruptly, leading to relatively large errors. For ease of analysis, the areal dust loading corresponding to the sharp rise in pressure drop during dust deposition is taken as the critical value for the onset of interpleat channel clogging, which falls within a range of ± 5 mg/cm² around the areal dust loading at the turning point.

To investigate the critical areal dust loading for interpleat channel clogging of filters, the pressure drop across the filter cartridge was measured at 1 mg/cm² increments of areal dust loading by adjusting the filtration time under identical experimental conditions. The filter was weighed before and after each test to calculate the actual areal dust loading, with the measurement error controlled within 5%. Each experiment was conducted in triplicate to ensure satisfactory repeatability.

**Fig. 11:**
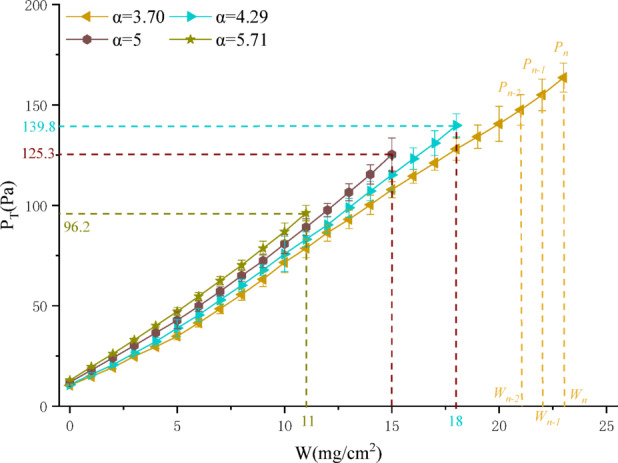
Critical areal dust loading for interpleat channel clogging of filters with 600-mesh fly ash at a filtration face velocity of 1 cm/s.

It is found that the growth rate ($\beta$) of the pressure drop variation corresponding to a 1 mg/cm² change in areal dust loading increases significantly when the interpleat channels of the filter cartridge are clogged. The formula for $\beta$ is given as follows:

1 $$\beta =\frac{{\left( {{P_n} - {P_{n - 1}}} \right) - \left( {{P_{n - 1}} - {P_{n - 2}}} \right)}}{{\left( {{P_{n - 1}} - {P_{n - 2}}} \right)}}$$

where ${P_n}$, ${P_{n - 1}}$and ${P_{n - 2}}$ denote the pressure drops across the filter cartridge at areal dust loadings of ${W_n}$, ${W_{n - 1}}$, and ${W_{n - 2}}$mg/cm^2^(Pa), respectively, as illustrated in Fig. 11.

**Fig. 12:**
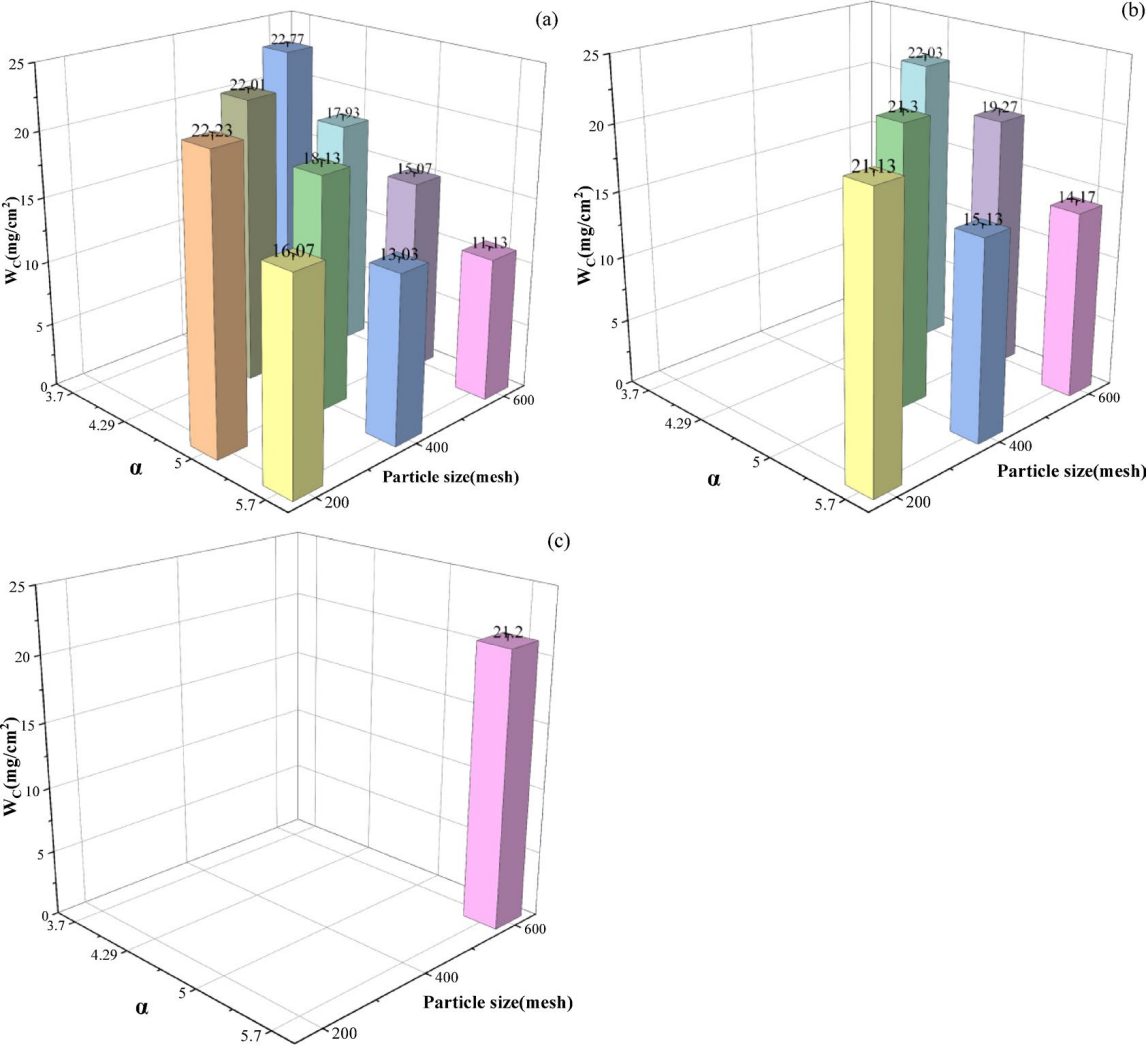
Critical areal dust loading (${W_C}$) for interpleat channel clogging of filters. **a** ${v_f}$=1 cm/s, **b** ${v_f}$=2 cm/s, **c** ${v_f}$=4 cm/s.

Data analysis shows that the value of $\beta$ is relatively small at low areal dust loadings, and increases gradually as dust deposition proceeds. As no clear demarcation exists for the occurrence of interpleat channel clogging, a threshold of $\beta =10\%$ is selected to determine the critical areal dust loading for clogging, according to the variation trend of filter pressure drop and the calculated values of B. If $\beta \geqslant 10\%$ appears within ± 5 mg/cm² of the areal dust loading at the turning point, the corresponding areal dust loading is identified as the critical value. At this point, the experiment is stopped and the filter cartridge is taken out for weighing. Figure 12 shows the critical value obtained for 600-mesh fly ash at a filtration velocity of 1 cm/s. Critical values under different experimental conditions can be obtained using the same method, as presented in Fig. 12.

A comparison of the critical areal dust loadings across distinct filtration face velocities demonstrates that interpleat channel clogging is most readily triggered at 1 cm/s, followed by 2 cm/s. By contrast, at a filtration face velocity of 4 cm/s, the critical loading is only detected for the filter cartridge with a pleat ratio of 5.71. Accordingly, the onset of interpleat channel clogging exhibits a clear dependence on filtration face velocity, with lower velocities facilitating clogging initiation. Figure 12(a) and (b) show that the critical areal dust loadings for interpleat channel clogging exhibit identical trends, decreasing overall with increasing pleat ratio and decreasing particle size. This indicates that a larger pleat ratio and smaller particle size render the filter cartridge more prone to interpleat channel clogging.

### Mechanism of interpleat channel blockage

Dust dendritic formation arises from the mutual adhesion of solid particles. The primary adhesive forces between solid particles include van der Waals forces, electrostatic attraction, and liquid bridge bonding forces, among others. Van der Waals forces act between molecules on solid surfaces and are much greater than gravitational forces for small particle sizes, allowing gravitational effects to be neglected. The van der Waals force between spheres of different sizes can be expressed as^21,22^:

2 $${F_{VW}}=\frac{A}{{12{l^2}}}\left( {\frac{{{D_{p1}}{D_{p2}}}}{{{D_{p1}}+{D_{p2}}}}} \right)$$

where ${F_{VW}}$ is the van der Waals force (N), *A* is the Hamaker constant of the particle in vacuum (an inherent material property, generally on the order of 10^− 19^ J), and ${D_{p1}}$ and ${D_{p2}}$ are the diameters of the two contacting spheres (m).

Solid particles acquire a net charge through processes such as mutual collision and friction. An electrostatic attractive force arises between two spheres bearing dissimilar charges. As investigated by Walton et al.^23^, the electrostatic attractive force dominates for small particle sizes and can play a significant role in particle adhesion. Since the corresponding effective energy is much higher than that of van der Waals interactions, these forces can significantly affect particle motion on surfaces and their final adhesion behavior.

**Fig. 13:**
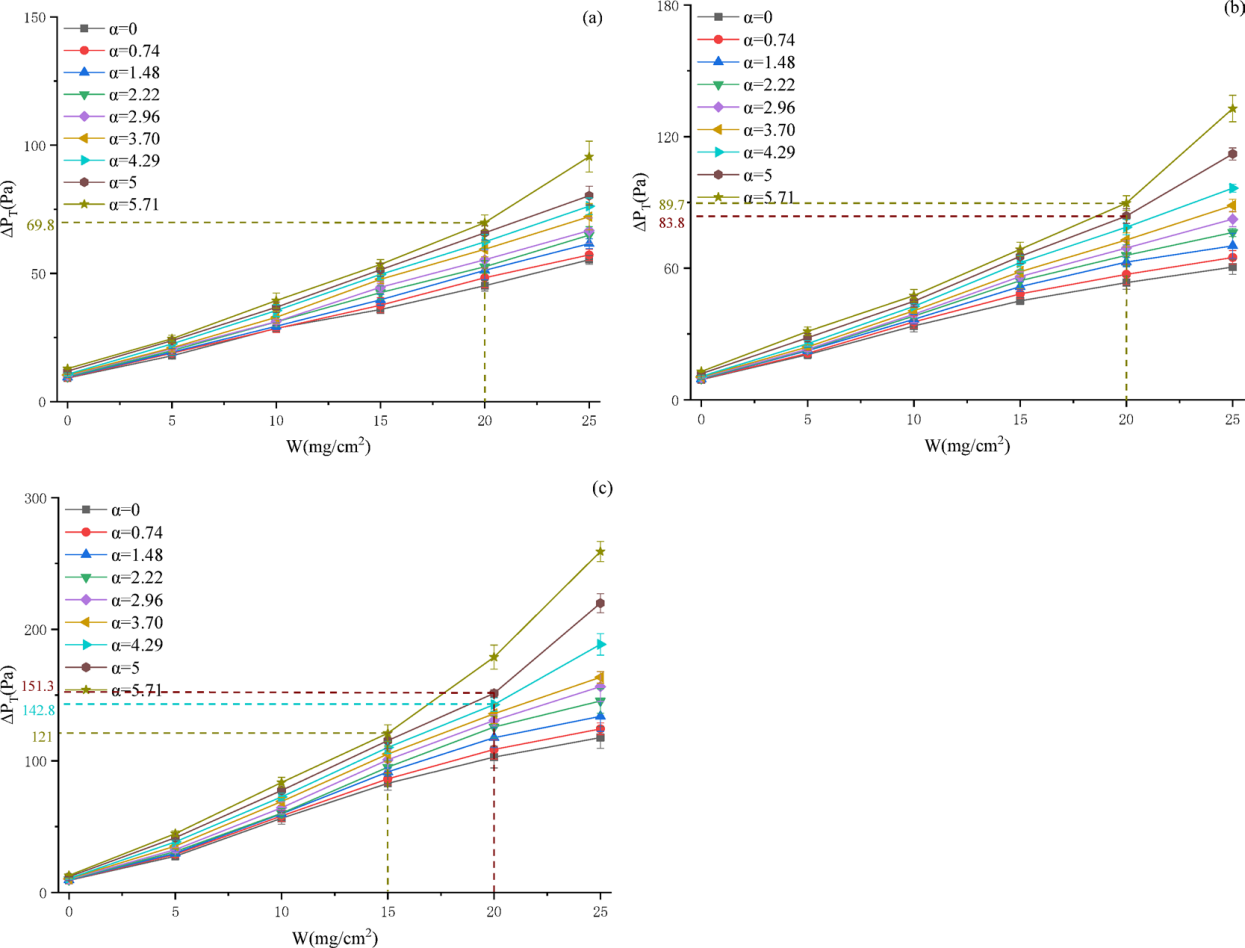
Variation of pressure drop of filter cartridge ($\Delta P$) with areal dust loading (*W*) at 40 ± 3% RH and a filtration face velocity of 1 cm/s **a** 200-mesh fly ash, **b** 400-mesh fly ash, **c** 600-mesh fly ash.

Owing to its small particle size, fly ash possesses a large specific surface area and pore volume, which enable it to adsorb moisture from the air. Meanwhile, some mineral components in fly ash exhibit strong hydrophilicity and can interact with water molecules, thereby further enhancing its hygroscopicity^24^. In this study, the experimental environment was maintained at an RH of 75 ± 5%, which may affect the adhesion behavior of dust particles. To examine the influence of RH on dust deposition, a series of comparative experiments were conducted at a filtration velocity of 1 cm/s with an RH of 40 ± 3%. The corresponding results are presented in Fig. 13.

A comparison with Fig. 8 reveals that when both the pleat ratio and areal dust loading are relatively low, the pressure drop remains essentially consistent, implying that RH has a negligible effect on pressure drop. However, at higher values, the pressure drop at 75 ± 5% RH is notably higher than that at 40 ± 3% RH, indicating that RH exerts a significant influence on pressure drop. The turning point of the filter pressure drop can be determined from the pressure drop variation, as illustrated in Fig. 13.

As illustrated in Fig. 13, during filtration of 200-mesh fly ash, an abrupt increase in pressure drop is observed at a pleat ratio of 5.71, with the corresponding areal dust loading at the turning point being 20 mg/cm². For 400-mesh fly ash, the areal dust loadings at the pressure drop turning points are both 20 mg/cm² at pleat ratios of 5 and 5.71. For 600-mesh fly ash, the corresponding values are 20, 20, and 15 mg/cm² at pleat ratios of 4.29, 5, and 5.71, respectively.

**Fig. 14:**
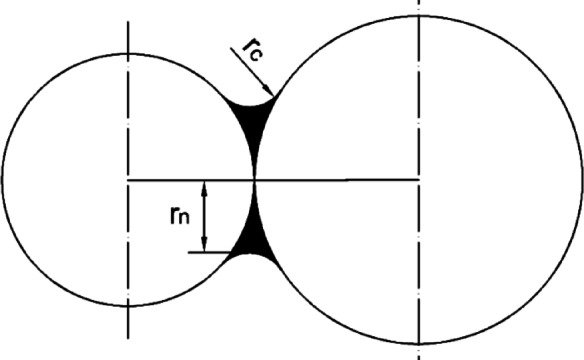
Schematic of interparticle liquid bridge formation.

By comparison with Fig. 8, it can be concluded that the filter cartridge is more susceptible to clogging at a relative humidity of 75 ± 5% RH, corresponding to a lower areal dust loading at the pressure drop turning point. In short, a higher RH accelerates the clogging of inter-pleat channels. This can be attributed to the fact that, under high-RH conditions, water vapor molecules in the air diffuse from the gas phase to the particle surfaces and infiltrate their internal micropores, where physical adsorption takes place and a thin water film forms on the particle surfaces^25–29^. On one hand, this thin water film enhances particle surface adhesion, promoting the adsorption of dust particles and the subsequent formation of dendritic structures. On the other hand, liquid bridges develop at the contact points between mutually adsorbed dust particles, producing liquid bridge cohesion that is substantially stronger than van der Waals forces and thus preventing the formed dust dendrites from fracturing easily^3,22^. The liquid bridge bonding force between two particles corresponds to the combined effect of capillary negative pressure and liquid surface tension *T*, as depicted in Fig. 14.

The computational expression for the liquid bridge bonding force between spheres of unequal diameters is given by^30^:

2 $${F_l}=\pi r_{n}^{2}T\left( {\frac{1}{{{r_c}}} - \frac{1}{{{r_n}}}} \right)+2\pi {r_n}T$$

Where ${F_l}$ is the liquid bridge bonding force (N), ${r_n}$ is the distance from the liquid surface to the line connecting the sphere centers (m), *T* is the liquid surface tension (N/m), and ${r_c}$ is the radius of curvature of the liquid (m).

Seville et al.^22^ and Renzo et al.^31^ observed that liquid bridge interactions become exceedingly strong for fine particles, greatly surpassing gravitational, van der Waals, and electrostatic forces. Furthermore, fine dust particles possess a markedly higher adsorption probability than their coarse counterparts, which arises from the enhanced interparticle forces at reduced particle sizes^32^. Consequently, fine dust particles readily adhere to one another under such interparticle actions, facilitating the formation of dust dendrites.

From Fig. 12, the critical areal dust loading for blockage of interpleat channels was obtained for the filter with a pleat ratio of 5.71. At a filtration face velocity of 1 cm/s, the critical areal dust loadings were 16.07, 13.03, and 11.13 mg/cm² for filtration of 200, 400, and 600 mesh fly ash, respectively; at 2 cm/s, the values were 21.13, 15.13, and 14.17 mg/cm², respectively; at 4 cm/s, a critical areal dust loading of 21.2 mg/cm² was only observed for filtration of 600 mesh fly ash. It can be seen that the critical areal dust loading for interpleat channel blockage generally decreases with decreasing dust particle size. Finer dust particles are more likely to form dust dendrites that block the interpleat channels, which is attributed to the stronger interparticle forces at smaller particle sizes.

Due to interparticle adhesion, abundant dust dendrites form in the interpleat channels during dust deposition, and these dendrites undergo repeated fracture and regeneration under the action of airflow forces. The aerodynamic force acting on a dust dendrite is dependent on its size and morphology. Bourrous et al.^11,33^ investigated the force characteristics of dust dendrites and expressed the applied force as:

3 $${\vec {F}_t}=\int {\int_{0}^{{{S_S}}} {Cv_{{loc}}^{2}} } dS$$

Where ${\vec {F}_t}$ is the airflow force acting on the dust dendrite (N), *C* is the airflow force coefficient (kg/m^3^), ${v_{loc}}$ is the velocity component perpendicular to the dust dendrite (m/s), ${S_S}$ is the projected area of the dust dendrite (m^2^).

From Eq. 3, the airflow force acting on the dust dendrite is shown to increase with flow velocity and its projected area. The corresponding shear stress can be expressed as:

4 $${R_t}=\frac{{{{\vec {F}}_t}}}{{{\operatorname{S} _{ec}}}}$$

where ${R_t}$ denotes the shear stress of the dust dendrite (N/m²) and ${S_{ec}}$ represents the cross-sectional area of the dust dendrite (m²).

From the above equations, a larger dust dendrite volume corresponds to a greater airflow force acting on the dendrite, which can induce dendrite fracture and thus restrict dendrite growth. Consequently, interpleat channel blockage only arises under small interpleat spacing. The relevant experimental data presented in the section "Study on influencing factors of dust clogging" of this paper verify this conclusion.

From Fig. 12, the critical areal dust loading for blockage of interpleat channels under different filtration face velocities can be obtained. Taking the critical areal dust loading during filtration of 600‑mesh fly ash as an example: at a pleat ratio of 5.71, the critical areal dust loadings were 11.13, 14.17, and 21.2 mg/cm² at filtration face velocities of 1, 2, and 4 cm/s, respectively. At a pleat ratio of 5, critical loadings were observed only at 1 and 2 cm/s, with values of 15.07 and 19.27 mg/cm², respectively. Similarly, at a pleat ratio of 4.29, critical loadings were also detected only at 1 and 2 cm/s, corresponding to 17.93 and 22.03 mg/cm², respectively. For a pleat ratio of 3.7, a critical areal dust loading of 22.77 mg/cm² was observed only at a filtration face velocity of 1 cm/s. It can be seen that a larger pleat ratio and a lower filtration face velocity favor the occurrence of the critical areal dust loading. This is attributed to the smaller dust dendrites formed, while higher filtration face velocity tends to induce their fracture.

## Conclusions

A systematic investigation was conducted via experimental research and theoretical analysis on the filtration performance of pleated filter elements with varying pleat ratios, as well as the governing factors for interpleat dust channel blockage.

The results demonstrate that pleating the filter medium reduces the effective filtration area and elevates the structural resistance. As a result, the filter resistance coefficient increases with the pleat ratio, and a higher pleat ratio leads to a larger pressure drop at identical areal dust loading. It is further observed that the pressure drop rises abruptly for filters with a greater pleat ratio under high dust loading conditions. This phenomenon arises from the formation of dust dendrites driven by the combined effects of van der Waals forces, electrostatic forces, and liquid bridge bonding forces, which subsequently induce interpleat channel blockage. In addition, the blockage propensity is enhanced with a larger pleat ratio, finer particle sizes and lower filtration face velocities. These findings provide important technical references for the structural design and engineering optimization of pleated filter elements in industrial applications.

## Data Availability

The datasets used and/or analyzed during the current study are available upon reasonable request from the corresponding author.
